# Supplementary material for: Detection and variability analyses of CRISPR-like loci in the H. pylori genome
Source: PeerJ. 2019 Jan 11;7:e6221. doi: 10.7717/peerj.6221 (PMC6330956; doi:10.7717/peerj.6221)
Supplement: Figure S1A — Cluster A including 25 strains. CRISPR-like locus of these strains showed four DRs and three spacers (as reference strain) with 86% of identity. The complete sequence of VlpC gene showed an 92% of identity. [file peerj-07-6221-s001.pdf]

Supplementary Figure S1. Analysis by cluster of the CRISPR-like loci inserted in the VlpC gene. The alignments of the VlpC gene using J99 strain as reference genome (first line) allowed us to classify all strains in six clusters attending to different structures found in DRs and spacers of CRISPR-like loci (A to F). Color indicates the degree of variation in both the gene and its CRISPR-like loci. Dark (high values of pairwise % identity), light (low pairwise % identity). Solid line indicates the presence of gaps. Alignment was performed with Muscle software. In the figures, only the region of gene that includes the CRISPR-like locus is showed.

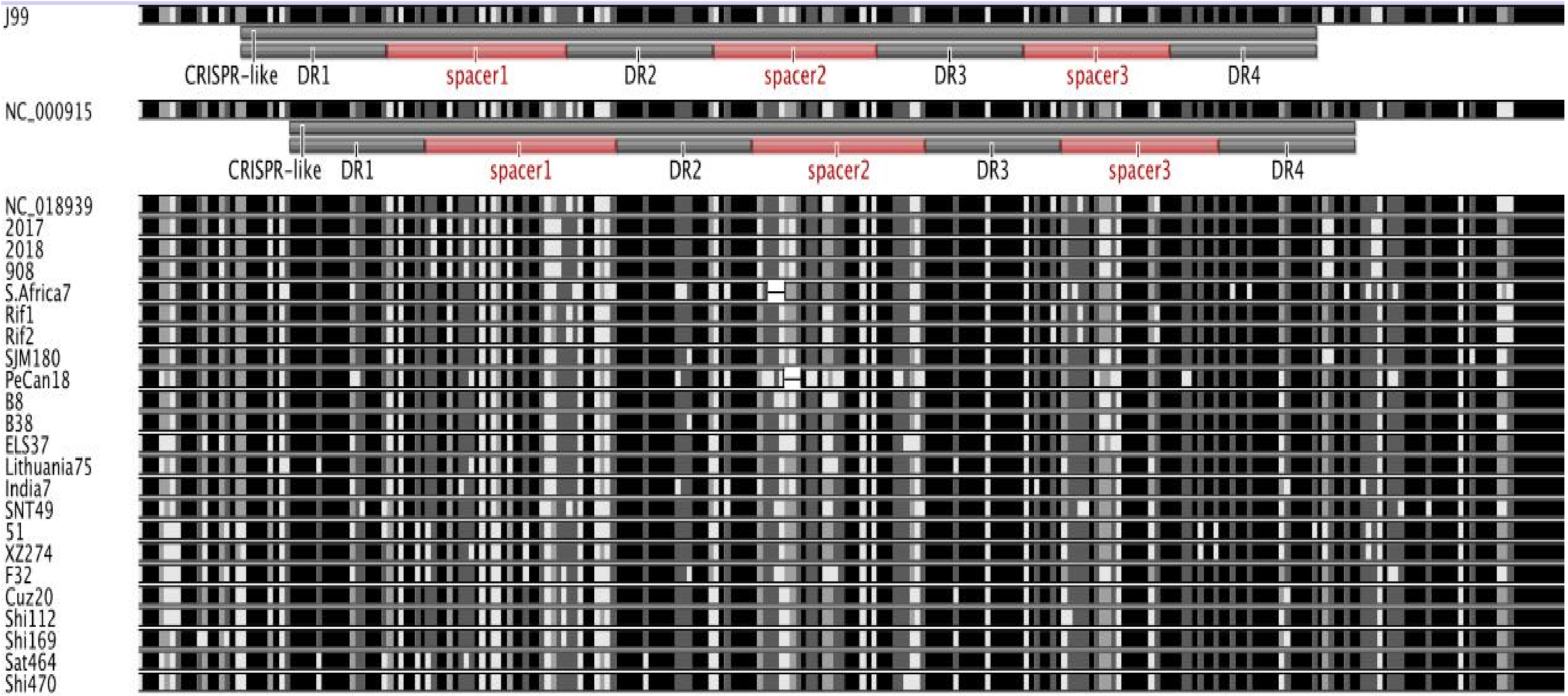
J99
CRISPR-like
DR1
spacer1
DR2
spacer2
DR3
spacer3
DR4
NC_000915
CRISPR-like
DR1
spacer1
DR2
spacer2
DR3
spacer3
DR4
NC_018939
2017
2018
908
S.Africa7
Rif1
Rif2
SJM180
PeCan18
B8
B38
ELS37
Lithuania75
India7
SNT49
51
XZ274
F32
Cuz20
Shi112
Shi169
Sat464
Shi470
