# Supplementary figures and images for: Detection and variability analyses of CRISPR-like loci in the H. pylori genome
Source: PeerJ. 2019 Jan 11;7:e6221. doi: 10.7717/peerj.6221 (PMC6330956; doi:10.7717/peerj.6221)

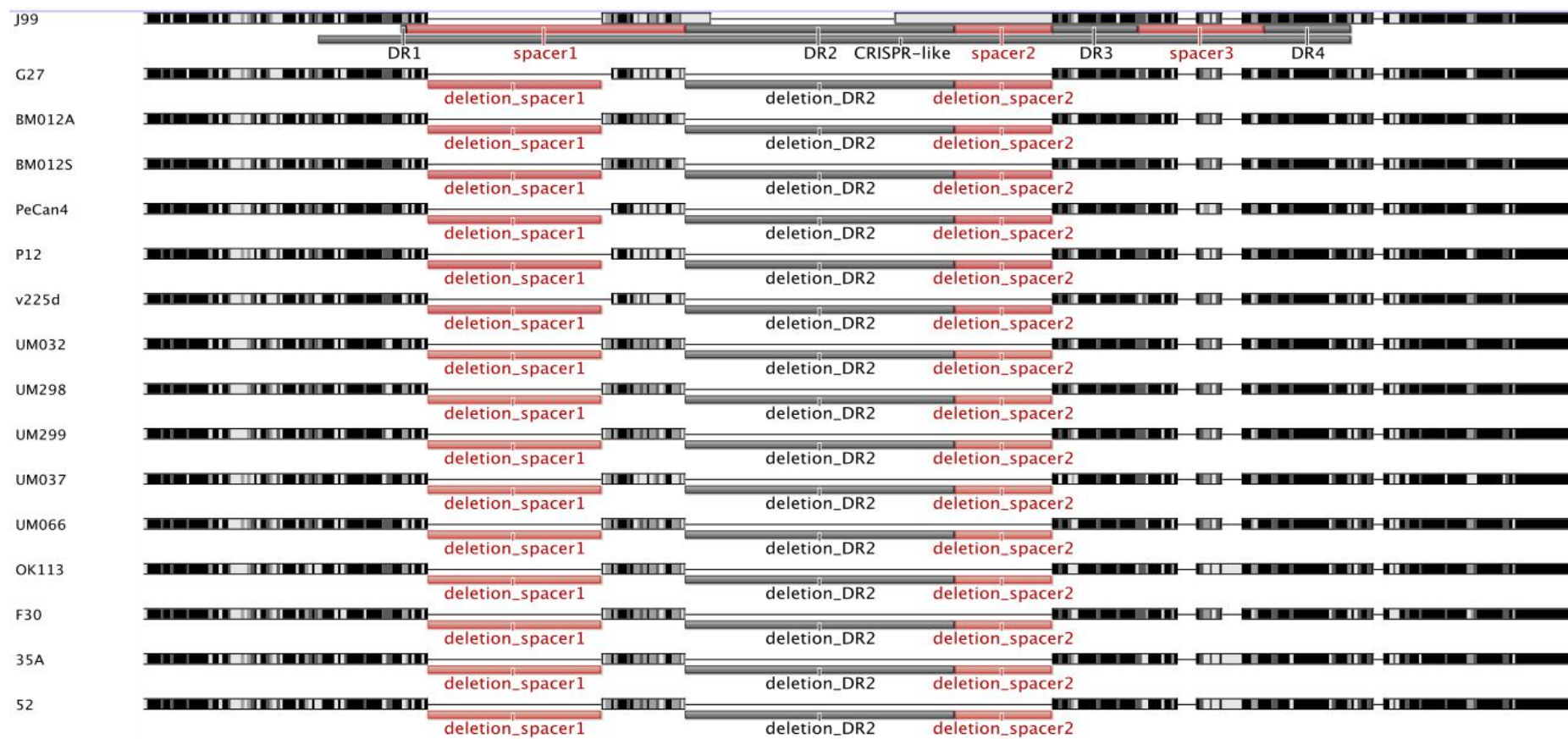

Supplement: Figure S1B — Cluster B including 15 strains. In these strains, two deletions are observed affecting the spacer1, DR2 and spacer2. Pairwise % identity in CRISPR-like loci and complete gene: 79% and 92%, respectively. [file peerj-07-6221-s002.pdf]

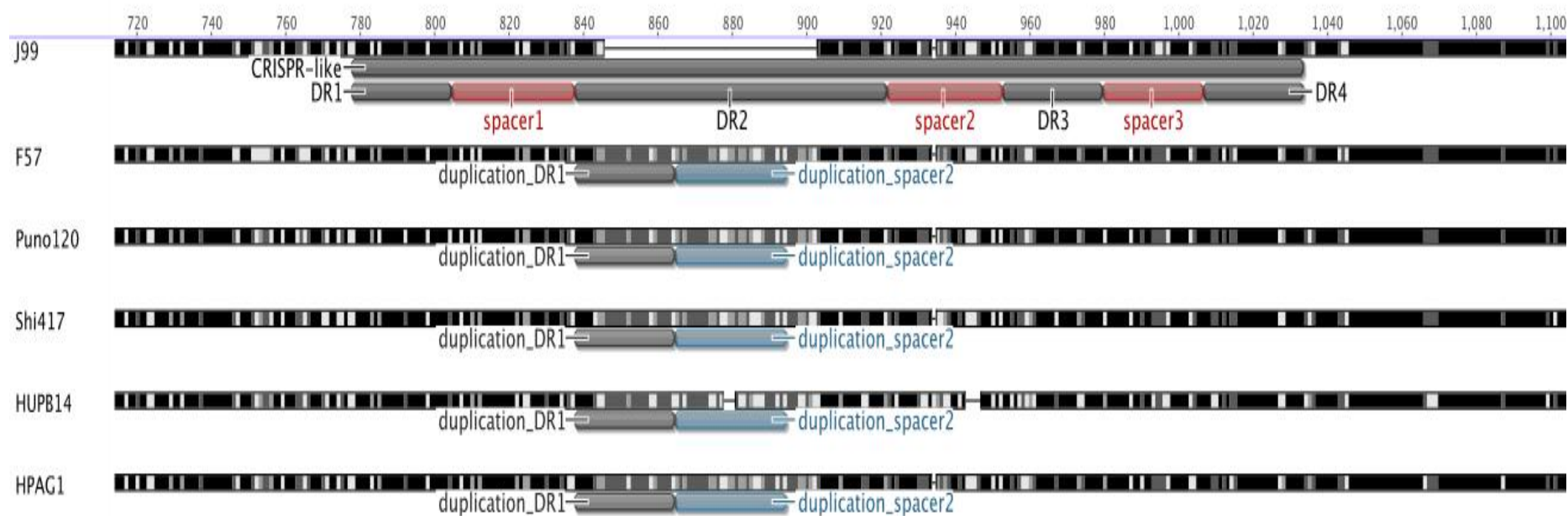

Supplement: Figure S1C — Cluster C including five strains. In these strains a duplication of DR2 and spacer2 is observed. Pairwise % identity in CRISPR-like loci and complete gene: 77% and 91%, respectively. [file peerj-07-6221-s003.pdf]

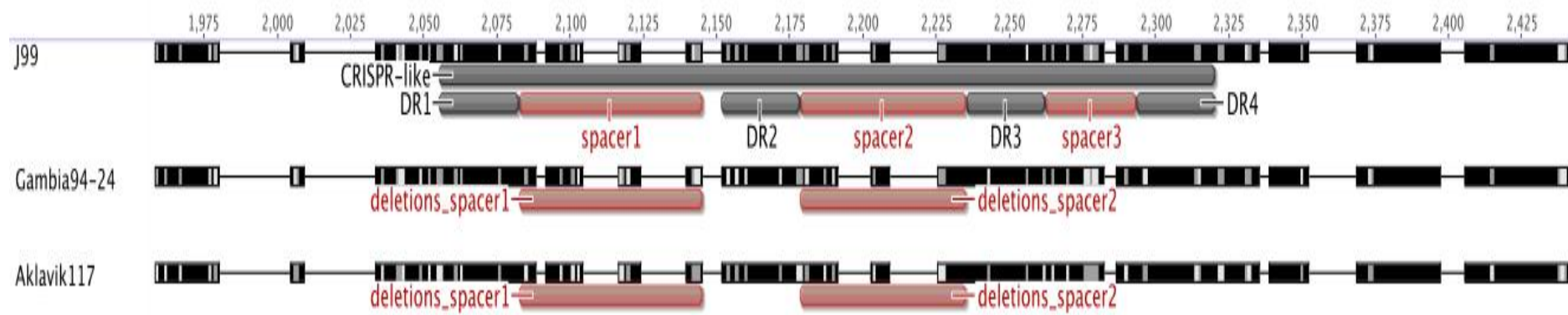

Supplement: Figure S1D — Cluster D including two strains. In these strains the spacers 1 and 2 are incomplete due to different deletions. Pairwise % identity in CRISPR-like loci and complete gene: 85% and 90%, respectively. [file peerj-07-6221-s004.pdf]

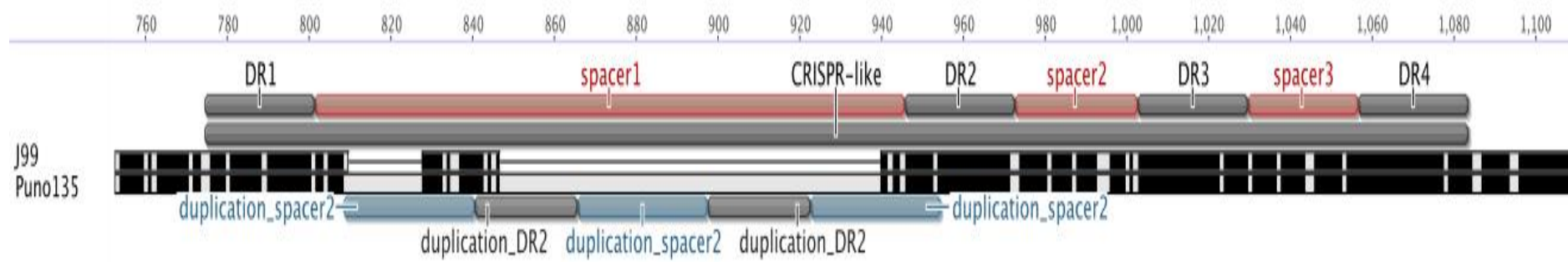

Supplement: Figure S1E — Cluster E including one strain which CRISPR-like sequence shows a deletion at the spacer1, two additional copies of DR2, and three additional copies of spacer2. Pairwise % identity in CRISPR-like loci and complete gene: 54% and 90%, respectively. [file peerj-07-6221-s005.pdf]

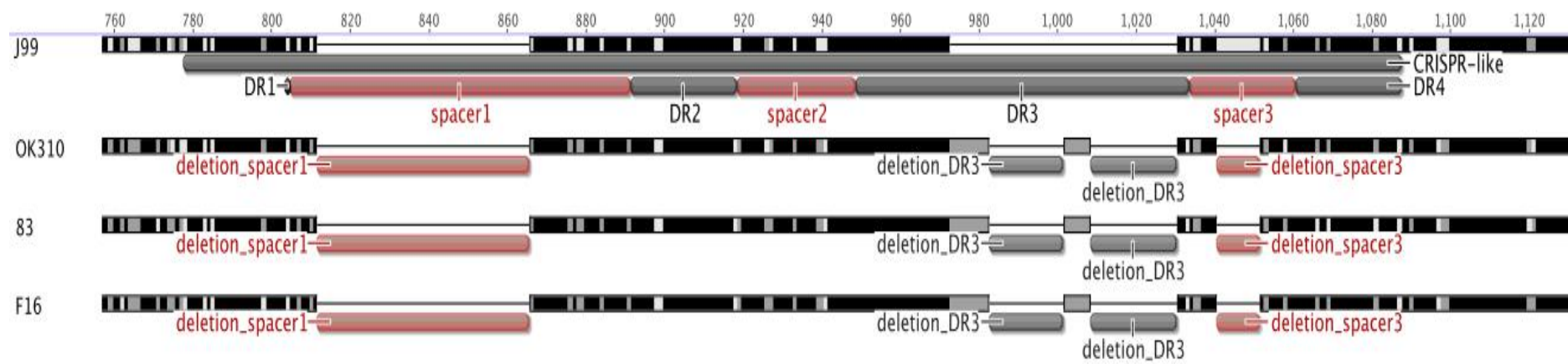

Supplement: Figure S1F — Cluster F including three strains. Their CRISPR-like sequences show deletion of the spacer1, and partials deletions at DR3 and spacer3. Pairwise % identity in CRISPR-like loci and complete gene: 85% and 94%, respectively. [file peerj-07-6221-s006.pdf]

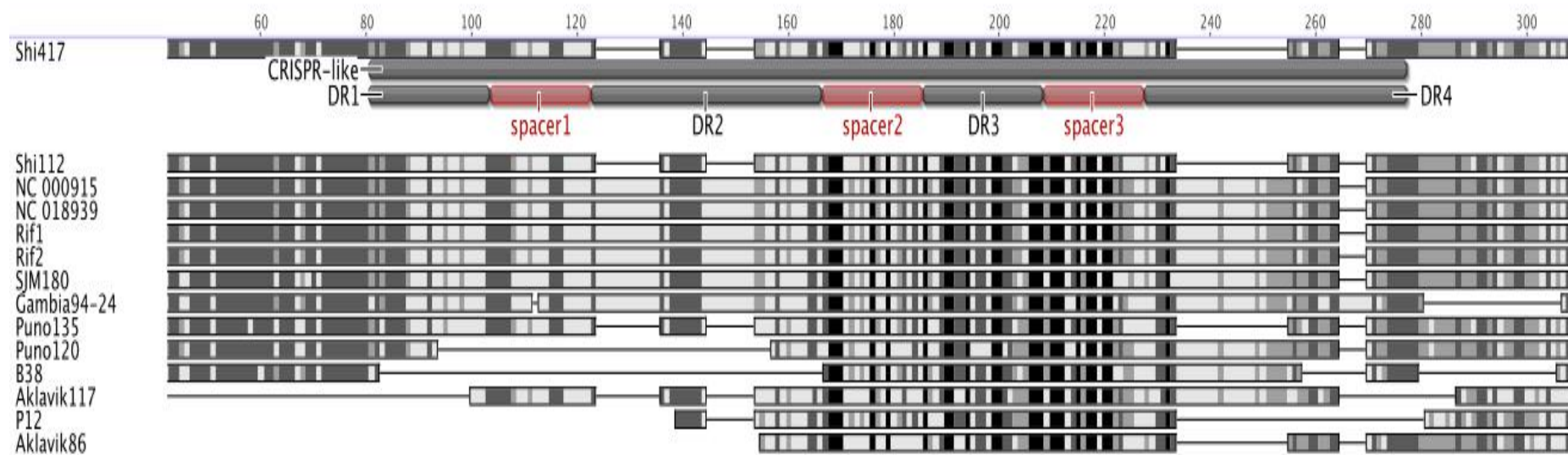

Supplement: Figure S2 — In the figure only the region of gene including CRISPR-like locus is showed. Color indicates the degree of variation in both the gene (hypothetical protein) and its CRISPR-like loci. Dark (high values of pairwise % identity), light (low pairwise % identity). Alignment was performed with Muscle software, using Shi417 as reference genome (first line). Repeated direct sequence (DR). Solid line indicates the presence of gaps. Pairwise % identity in CRISPR-like loci: 56%, and in the complete gene: 72%. Three strains (Aklavik86, Aklavik117 and P12) showed the 5’ region truncated. [file peerj-07-6221-s007.pdf]

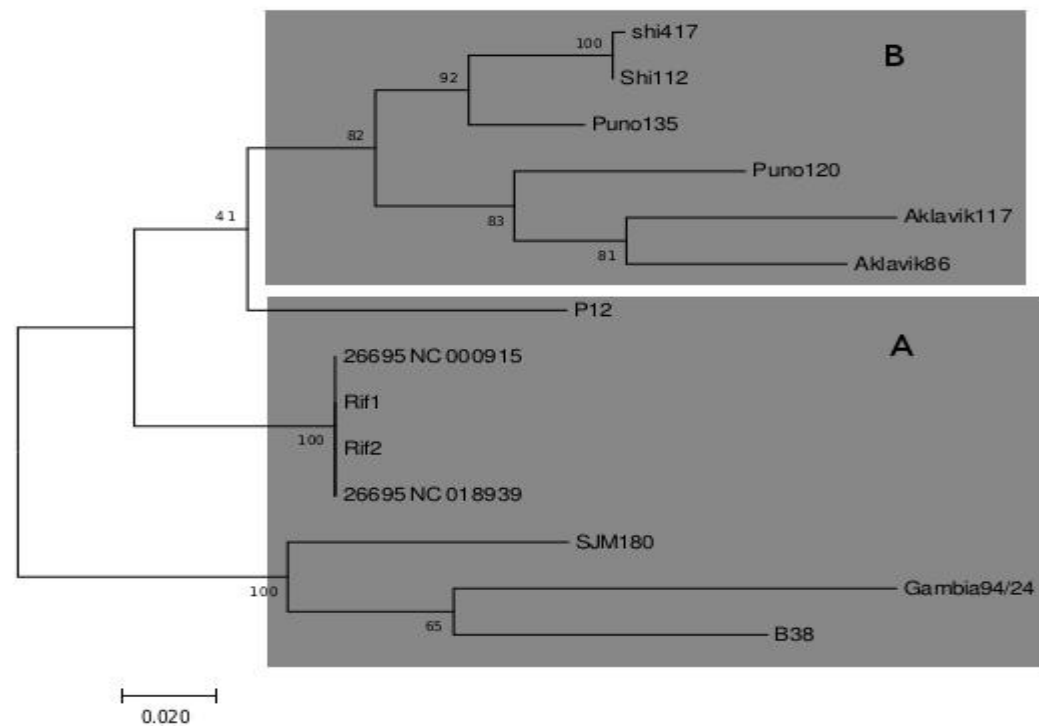

Supplement: Figure S3 — A phylogeographic differentiation of CRISPR-like loci is observed. Analysis performed using MEGA7 software. Evolutionary distance scale: 0.02 model of Jukes-Cantor. (A) Group of African and European geographical origin. (B) Amerind geographic group. [file peerj-07-6221-s008.pdf]

A

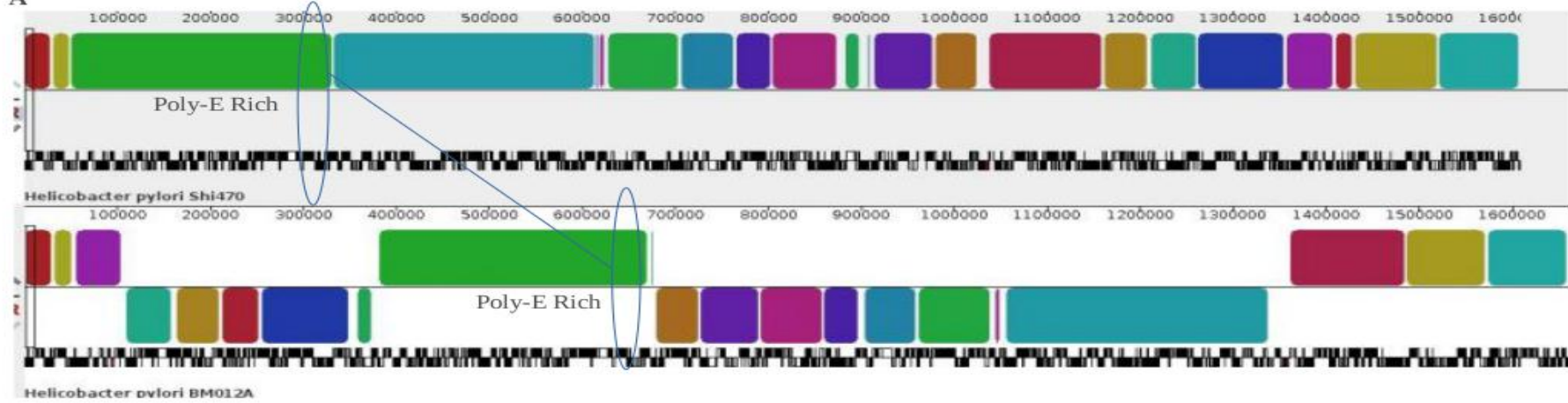

B

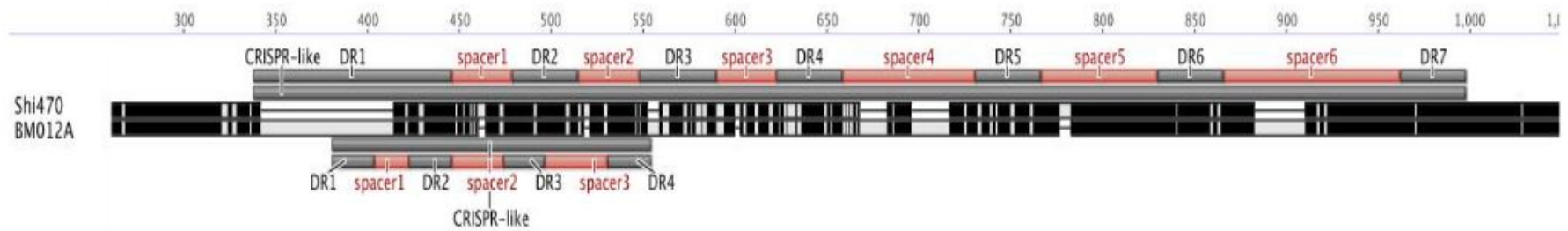

Supplement: Figure S4 — (A) Alignment of the Shi470 and BM012A genomes using Mauve software. Figures show the genome region where Poly-E rich protein gene is included. The alignment revealed that this gene was in a region close to the breaking point of an inversion that affects these strains (blue oval). (B) Alignment of the gene for the Poly-E rich protein. Color indicates the degree of variation in both the gene and its CRISPR-like loci. Dark (high values of pairwise % identity), light (low pairwise % identity). Alignment was performed with Muscle software. Repeated direct sequence (DR). Solid line indicates the presence of gaps. The alignment suggests that the differences observed can be explained by the number of DR sequences and spacers in which they differ. [file peerj-07-6221-s009.pdf]

Shi470 Poly E Rich

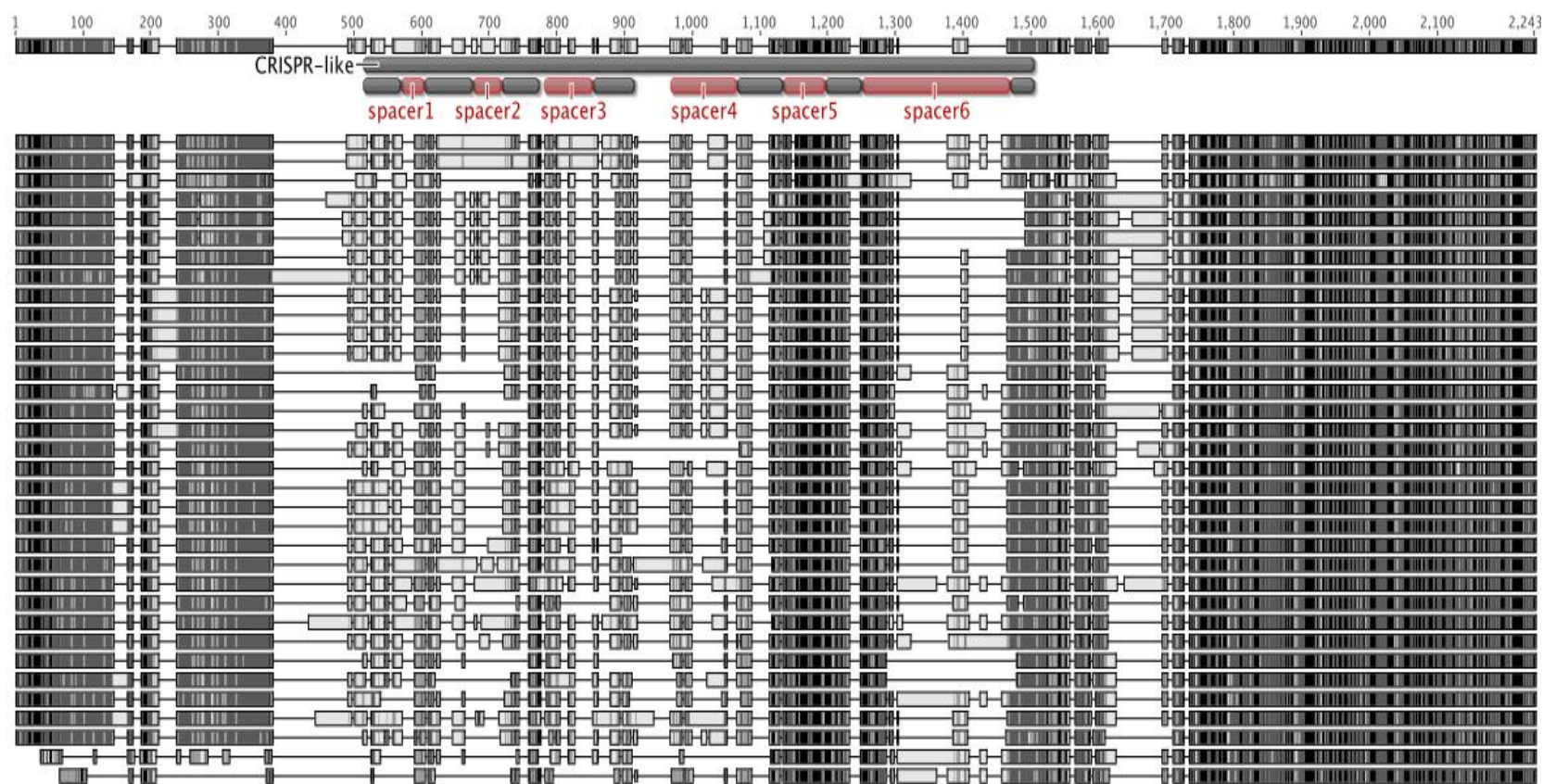

Supplement: Figure S5 — Color indicates the degree of variation in both the Poly-E rich protein gene and its CRISPR-like loci. Dark (high values of pairwise % identity), light (low pairwise % identity). Alignment was performed with Muscle software, using Shi470 strains as reference genome (first line). Repeated direct sequence (DR). Solid line indicates the presence of gaps. The alignment revealed a middle location of the CRISPR-like loci, showing a high variability for this region. Pairwise % identity in CRISPR-like loci: 60%, and in the complete gene: 80%. [file peerj-07-6221-s010.pdf]

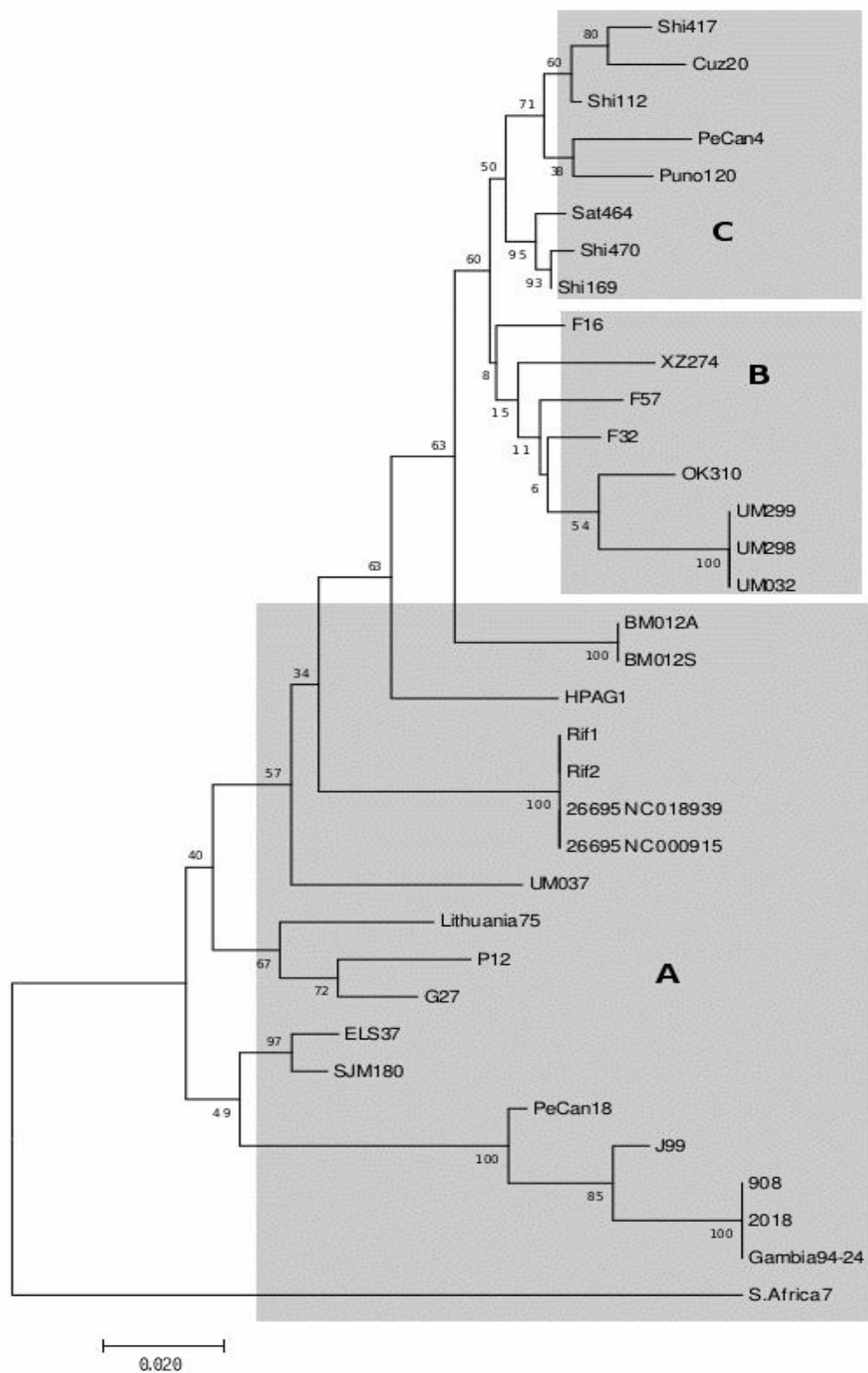

Supplement: Figure S6 — A phylogeographic differentiation of CRISPR-like loci is observed. Analysis performed using MEGA7 software. The evolutionary distance scale : in 0.02. Neighbor-joining method with bootstrap of 1,000 replications and Jukes-Cantor model. (A) Group of African and European geographical origin. (B) Asia geographic group. (C) Amerind group. [file peerj-07-6221-s011.pdf]

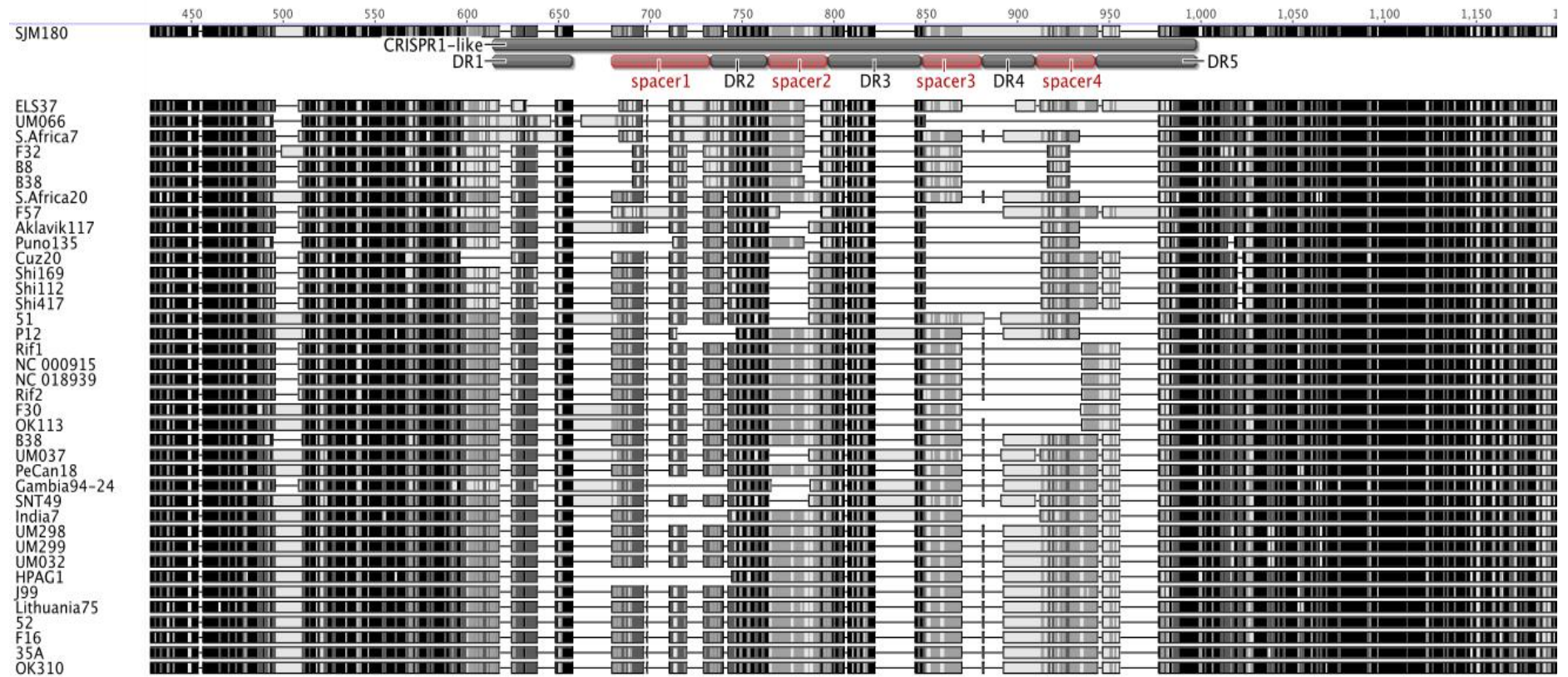

Supplement: Figure S7 — Color indicates the degree of variation in both the hypothetical protein gene and its CRISPR-like loci. Dark (high values of pairwise % identity), light (low pairwise % identity). Alignment was performed with Muscle software, using SJM180 strain as reference genome (first line). Repeated direct sequence (DR). Solid line indicates the presence of gaps. The alignment revealed that the gene of the CRISPR1-like loci occupies a central position. In the alignment, degeneration of CRISPR1-like loci can be observed in contrast to high degree of conservation of 5 ’and 3’ regions. Pairwise % identity in CRISPR-like loci and complete gene: 63% and 85%, respectively. [file peerj-07-6221-s012.pdf]

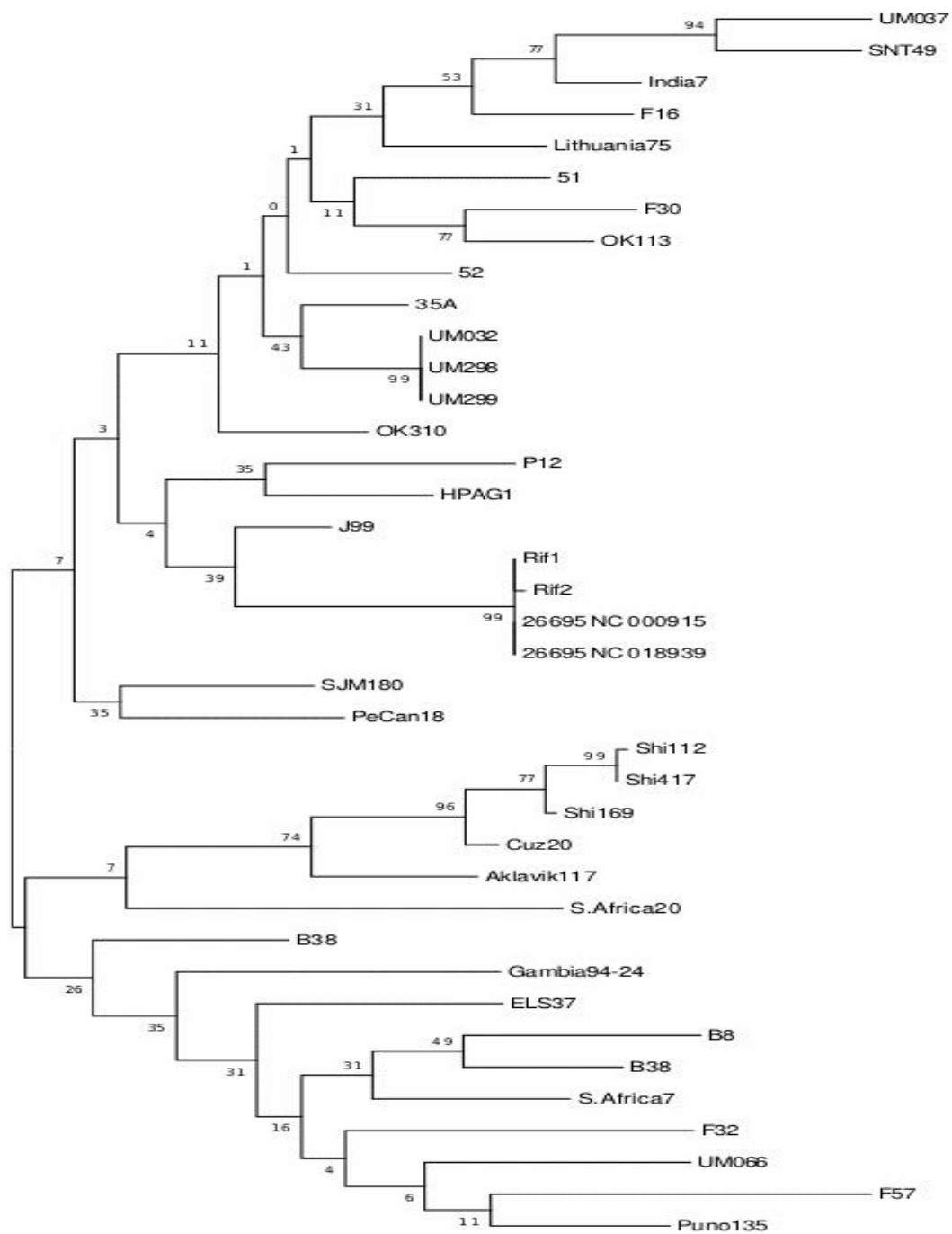

0.0100

Supplement: Figure S8 — Phylogeographic differentiation of CRISPR-like loci is not observed. Analysis was performed using MEGA7 software. The evolutionary distance scales in 0.01. Neighbor-joining method with bootstrap of 1,000 replications and Jukes-Cantor model. [file peerj-07-6221-s013.pdf]

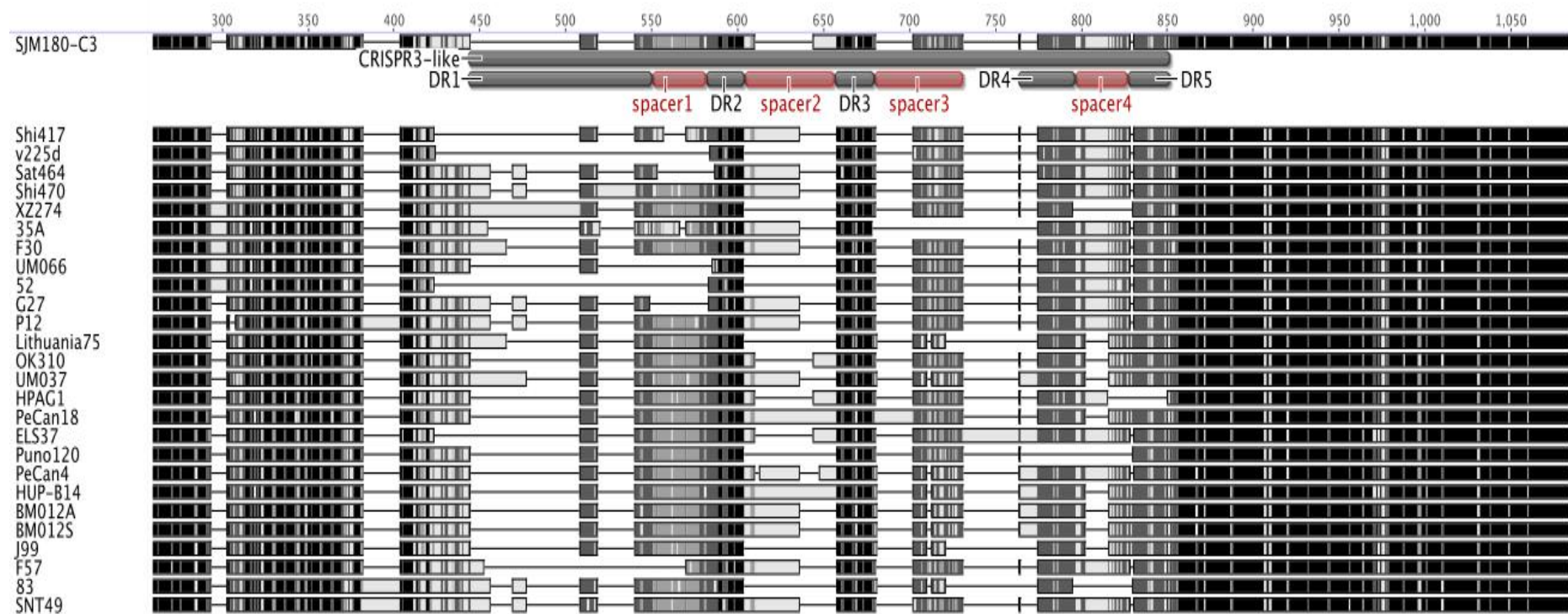

Supplement: Figure S9 — Color indicates the degree of variation in both the hypothetical protein gene and its CRISPR-like loci. Dark (high values of pairwise % identity), light (low pairwise % identity). Alignment was performed with Muscle software, using SJM180 strain as reference genome (first line). Repeated direct sequence (DR). Solid line indicates the presence of gaps. The alignment revealed that CRISPR3-like loci occupies a central position. In the alignment, degeneration of CRISPR1-like loci can be observed in contrast to high degree of conservation of 5’ and 3’ regions. Pairwise % identity in CRISPR-like loci and complete gene: 61% and 84%, respectively. [file peerj-07-6221-s014.pdf]

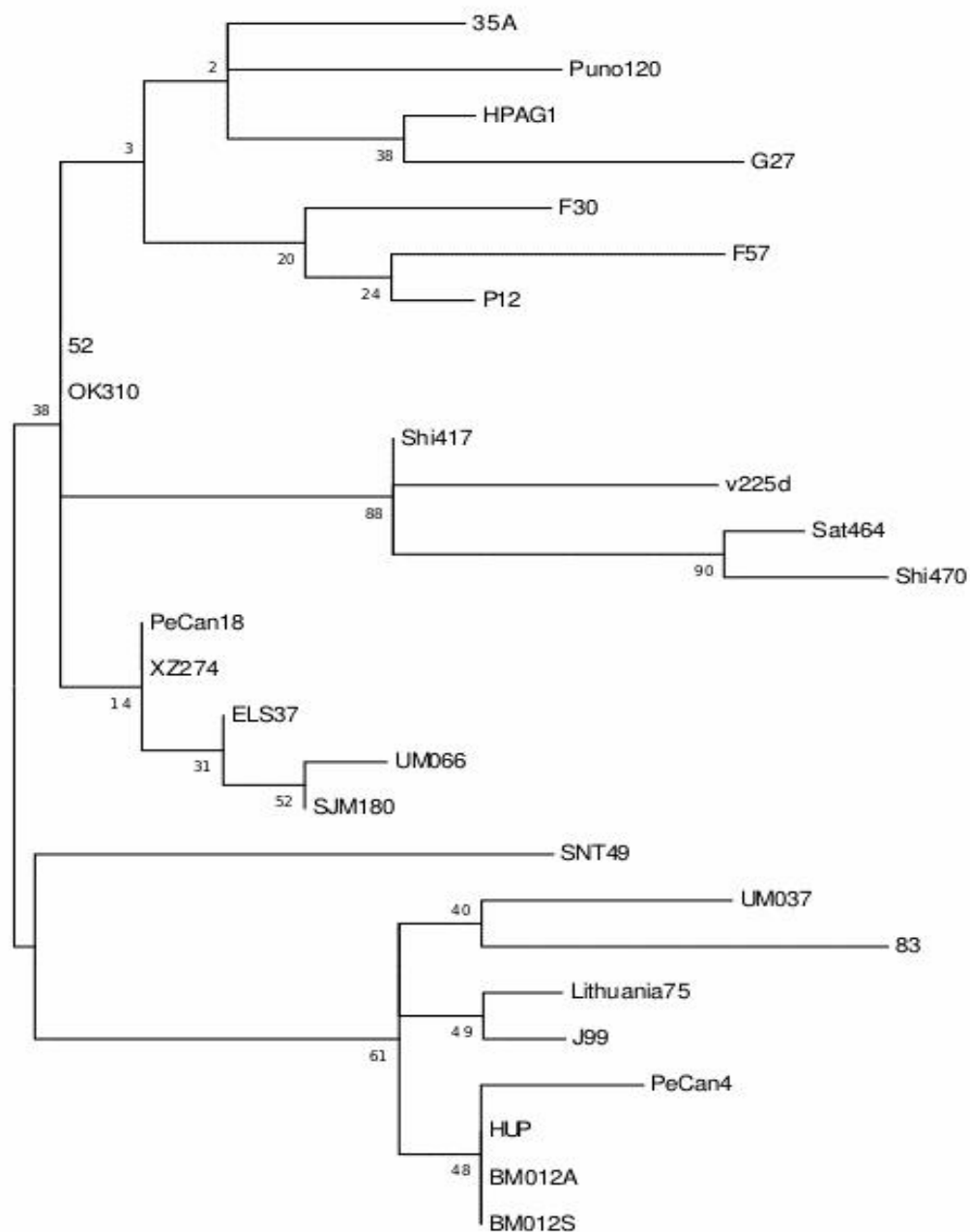

0.0100

Supplement: Figure S10 — Phylogeographic differentiation of CRISPR-like loci is not observed. Analysis was performed using MEGA7 software. The evolutionary distance scales in 0.01. Neighbor-joining method with bootstrap of 1,000 replications and Jukes-Cantor model. [file peerj-07-6221-s015.pdf]
