# Supplementary material for: Detection and variability analyses of CRISPR-like loci in the H. pylori genome
Source: PeerJ. 2019 Jan 11;7:e6221. doi: 10.7717/peerj.6221 (PMC6330956; doi:10.7717/peerj.6221)
Supplement: Figure S11 — The analysis of DRs using RNAfold Server allowed us to predict the secondary structure of RNAs and record their minimum free energy (MFE). Consensus sequence was used for each cluster (described in Suplementary Figure S1, Figure S2 and Figure S5). In all clusters, RNA secondary structure showed central stems with joined ends as shown in the figure. The stability of the sequence was predicted considering the MFE value, and the fluctuation between −0.74 kcal / mol and −7.73 kcal / mol. Structures with lower MFE are presumably the most stable. [file peerj-07-6221-s016.pdf]

Direct Repeat Sequences (DRs)

Cluster 1

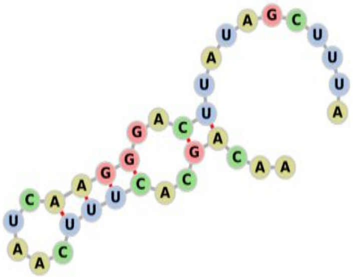

-1.73 kcal/mol

Cluster 2

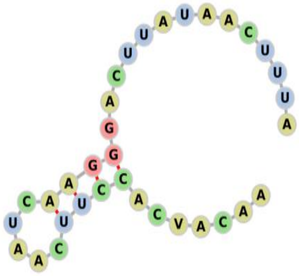

-2.73 kcal/mol

Cluster 3

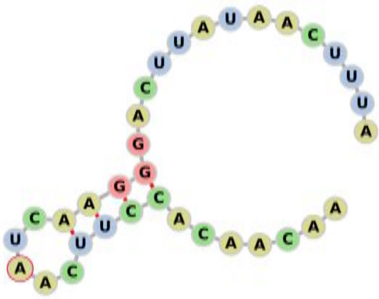

-2.75 kcal/mol

Cluster 4

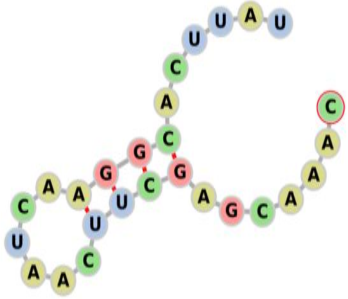

-2.14 kcal/mol

Cluster 5

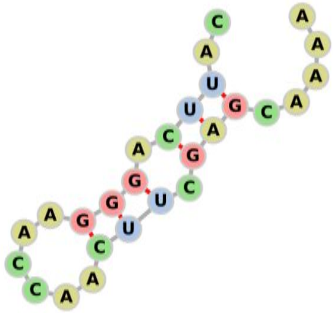

-4.02 kcal/mol

Cluster 6

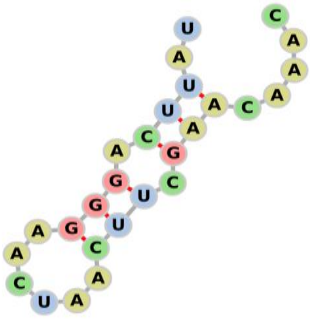

-2.98 kcal/mol

BM012A (Poly E rich protein)

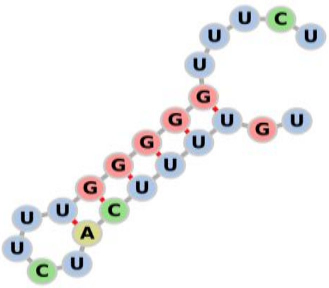

-1.59 kcal/mol

Shi470 (Poly E rich protein)

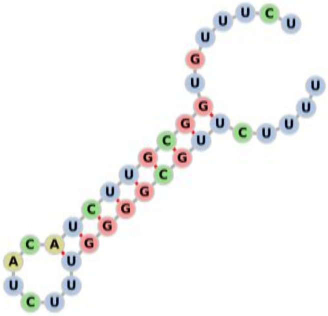

-7.73 kcal/mol

Shi417 (Hypothetical protein)

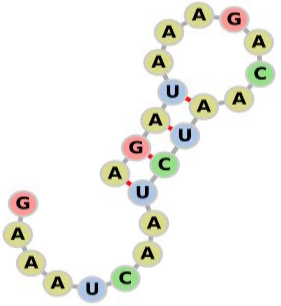

-0.74 kcal/mol

Shi112 (Hypothetical protein)

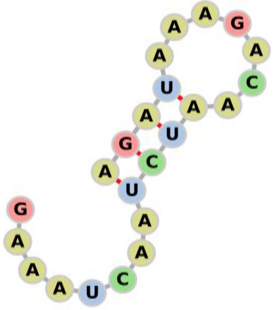

-0.74 kcal/mol
