# Supplementary material for: Detection and variability analyses of CRISPR-like loci in the H. pylori genome
Source: PeerJ. 2019 Jan 11;7:e6221. doi: 10.7717/peerj.6221 (PMC6330956; doi:10.7717/peerj.6221)
Supplement: Table S1 — Characteristics of the repeated direct sequences (DRs) consensus and of the spacer sequences of the 22 CRISPR-like identified with CRISPRFinder [file peerj-07-6221-s017.docx]

| **Strains** | **DRs of the length (bp)** | **sequence DR** | **DRs number in CRISPR locus** | **Spacers number** | **Length of spacers (bp) present in the CRISPR-like loci** |
| --- | --- | --- | --- | --- | --- |
| NC_000915 | 25 | TTCAATCAAGGGACTTATCACTTTA | 4 | 3 | 35, 32, 29 |
| HPAG1 | 28 | TTTCAATCAAGGGACTTATAACTTTAAT | 5 | 4 | 32, 29, 29, 26 |
| B8 | 25 | TTCAATCAAGGGACTTATCACTTTA | 4 | 3 | 35, 32, 29 |
| B38 | 25 | TTCAATCAAGGGACTTATCACTTTA | 4 | 3 | 35, 32,29 |
| Rif1 | 25 | TTCAATCAAGGGACTTATCACTTTA | 4 | 3 | 35, 32, 29 |
| Rif2 | 25 | TTCAATCAAGGGACTTATCACTTTA | 4 | 3 | 35, 32, 29 |
| NC_018939 | 25 | TTCAATCAAGGGACTTATCACTTTA | 4 | 3 | 32, 29, 29, 26 |
| 2017 | 27 | TTCAATCAAGGCACTTATAATTTTAAT | 4 | 3 | 33, 30, 27 |
| 2018 | 27 | TTCAATCAAGGCACTTATAATTTTAAT | 4 | 3 | 33, 30, 27 |
| 908 | 27 | TTCAATCAAGGCACTTATAATTTTAAT | 4 | 3 | 33, 30, 27 |
| XZ274 | 23 | AACTTCAATCAAGGGACTTATAA | 4 | 3 | 22, 49, 26 |
| India7 | 25 | TTCAATCAAGGCACTTATCATTTTA | 4 | 3 | 35, 32, 29 |
| SNT49 | 27 | TTCAATCAAGGGACTTATCATTTTAAT | 4 | 3 | 33, 30, 27 |
| F57 | 26 | TTTCAATCAAGGGACTTATAATTTTA | 5 | 4 | 34, 31, 28, 28 |
| J99 | 27 | AACAGCAATTTCAATCAAGGGACTTAT | 4 | 3 | 33, 30, 27 |
| Shi112 | 23 | AGATAAAGACAATCTAACTAAAG | 4 | 3 | 19, 19, 19 |
| Shi417 | 23 | AGATAAAGACAATCTAACTAAAG | 4 | 3 | 19, 19, 19 |
| Shi470 | 36 | TTTTCTTGCGGGGTTTCTACATCTTGCGGTGTTTCT | 7 | 6 | 33, 33, 33, 33, 36, 63, 69 |
| SJM180 | 29 | TCTTGTTTGTTTTCTGTCTTTCTTGTTCT | 5 | 4 | 34, 34, 34, 34 |
| SJM180 | 25 | TTCAATCAAGGGACTTATCACTTTA | 4 | 3 | 35, 32, 29 |
| SJM180 | 23 | TTGTCTTTTGTTTTTCTTGTTCT | 5 | 4 | 31, 19, 31, 31 |
| BM012A | 23 | TTTCTACATCTTGCGGTATTTCT | 4 | 2 | 19, 25, 31 |
